# The Good Start Matters mHealth Parenting Program: Protocol for a Randomized Controlled Trial

**DOI:** 10.2196/72642

**Published:** 2025-10-23

**Authors:** Olivia De-Jongh González, Claire N Tugault-Lafleur, Janet W T Mah, Louise C Mâsse

**Affiliations:** 1 School of Population and Public Health, Faculty of Medicine BC Children’s Hospital Research Institute University of British Columbia Vancouver, BC Canada; 2 School of Nutrition Sciences, Faculty of Health Sciences University of Ottawa Ottawa, ON Canada; 3 Department of Psychiatry BC Children’s Hospital Research Institute University of British Columbia Vancouver, BC Canada

**Keywords:** parenting practices, coparenting, child health behaviors, mHealth, lifestyle intervention

## Abstract

**Background:**

The family plays a critical role in shaping children’s health behaviors during early childhood. Family-based interventions are a cornerstone of childhood obesity prevention but often yield modest effects and have several limitations, including a focus on a single caregiver and insufficient attention to coparenting dynamics. Mobile health (mHealth) interventions that include multiple caregivers and target coparenting practices are rare, but have the potential to amplify parenting intervention effects, leading to stronger child health outcomes.

**Objective:**

This protocol aims to describe a randomized controlled trial designed to evaluate the efficacy of the Good Start Matters mHealth Parenting Program in improving parenting and coparenting practices (primary outcomes) and child health behaviors (secondary outcomes) among 2.5- to 6-year-olds.

**Methods:**

This randomized controlled trial will recruit 118 two-parent families (ie, families with 2 caregivers participating in the trial) from childcare centers across British Columbia, Canada. To promote inclusivity, one-parent families (ie, only one parent participating) will also be eligible but will not count toward the target sample size for recruitment purposes. Eligibility criteria include at least one parent having primary custody of a child aged 2.5-6 years, both parents being fluent in English, and each caregiver owning a smartphone device. In addition, children must not have severe limitations that prevent adherence to general nutritional and 24-hour Movement Guidelines, nor can they be undergoing weight management treatment. After baseline data collection, families will be randomized into either the intervention group, which will receive immediate access to the app, or a waitlist control group, which will gain access to the app after the follow-up assessment, approximately 2 months later. Baseline and follow-up assessments will collect data on food, physical activity, and media parenting practices, coparenting agreement, and child eating, active play, and screen time behaviors. To analyze the data while accounting for its nested structure, multilevel mixed-effects models, integrating intention-to-treat principles and imputation techniques when deemed necessary, will be used. Sensitivity dose-response analyses will assess the extent to which differential adherence/exposure to the intervention influences the study’s outcomes.

**Results:**

The trial was registered in March 2023. Recruitment took place from November 2023 to January 2025, with 121 two-parent families and 62 one-parent families recruited. Data collection took place between November 2023 and April 2025. Data cleaning and analyses started in May 2025. Results are expected to be published in 2026.

**Conclusions:**

This study will provide critical insights into the efficacy of mHealth interventions to improve parenting and coparenting practices while promoting healthier child behaviors during early childhood. The Good Start Matters mHealth Parenting Program has the potential to strengthen the foundation of family-centered interventions and set a new standard for systemic approaches to early childhood obesity prevention.

**Trial Registration:**

ClinicalTrials.gov NCT05802160; https://clinicaltrials.gov/study/NCT05802160

**International Registered Report Identifier (IRRID):**

DERR1-10.2196/72642

## Introduction

### Background

Early childhood is a formative period for developing eating, activity, and screen-time habits, which significantly shape long-term health outcomes, including obesity risk [1,2]. Research [3-11] indicates that the family environment plays a critical role in shaping children’s health behaviors through their parenting practices; the goal-oriented strategies parents use to guide their child’s behaviors in a given context, such as nutrition or physical activity [7]. Parenting practices are typically categorized into 3 domains [7]: control (coercive and intrusive strategies to manipulate the child’s behaviors and emotions); structure (noncoercive practices that organize the child’s environment to support healthy habits); and autonomy promotion (strategies that encourage healthy habits while fostering independence and psychological autonomy). Structured and autonomy-promoting practices are associated with healthier diets, higher physical activity levels, and lower screen-time, whereas controlling practices are linked to unhealthy habits and increased obesity risk [3,5,6,8-10].

Despite the well-established influence of parenting practices, key limitations remain in our understanding of the family’s role in childhood obesity. First, most studies have focused on a single caregiver (typically the mother) due to maternal predominance in child-rearing and difficulties in recruiting and engaging fathers [11-14]. However, emerging evidence underscores the distinct and complementary roles that mothers and fathers play in shaping child health behaviors. Studies examining feeding, physical activity, and media parenting suggest that mothers and fathers may interact with children in unique ways [3,6,15], potentially offering additive benefits when both caregivers are engaged in interventions.

Second, few studies have considered the coparenting subsystem [16]; that is, how caregivers collaborate in child-rearing and their agreement on parenting strategies, division of labor, and joint family management [17]. While underresearched, evidence suggests that low-quality coparenting (eg, undermining and inconsistent coparenting) can compromise parenting practices and is associated with negative child health outcomes, including increased picky eating, frequent snacking, and obesity [18-22]. Another study shows that children are more likely to meet physical activity guidelines when both parents support active behaviors [23].

Given the critical role of the family system, family-based lifestyle interventions are a cornerstone of childhood obesity prevention and treatment [24-26]. However, in-person programs have modest effects [24], likely due to the aforementioned limitations and practical barriers to participation, such as scheduling constraints and low caregiver engagement. Consequently, digital health interventions, particularly mobile health (mHealth) apps, are increasingly being used to support parenting and promote healthy behaviors in children. mHealth platforms offer advantages over traditional delivery formats, including accessibility, cost-effectiveness, scalability, and real-time feedback to support participant engagement [27,28]. These features could potentially help overcome persistent challenges in family-based interventions, such as reaching multiple caregivers. A 2023 integrative review [29] found that parents valued the flexibility and convenience of digital delivery, especially the ability to participate from home at times that suited their busy schedules.

Recent systematic reviews and meta-analyses [30-33] have shown promising, yet mixed, outcomes for mHealth interventions targeting childhood obesity. Programs grounded in behavioral theory, particularly social cognitive theory (SCT) and self-determination theory, and incorporating diverse behavior change techniques (BCTs), such as goal setting, feedback, monitoring, and social support, are more effective [32-34].

However, important gaps persist as most mHealth interventions focus on school-aged children and adolescents, with limited attention to the early years (2-5 years) [27,32,35]. When younger children are included in such interventions, weaker effects emerge among them, particularly for physical activity [32,33], possibly due to greater reliance on childcare providers. This highlights the need for early childhood interventions that explicitly target parenting practices. Surprisingly, parenting practices are rarely primary outcomes in these interventions, which often focus on more distant health outcomes such as child behaviors and weight [27,30-32,34-36], thereby overlooking the parenting strategies that influence them. Moreover, when parenting practices are targeted, the emphasis is on controlling practices, with less attention to positive parenting practices such as structure and autonomy promotion, and with effects that often diminish over time [31]. To address these shortcomings, Wang et al [31] have called for future trials to target a broader range of parenting practices. We further suggest that intervention effects may be strengthened by supporting a more unified caregiving approach. However, coparenting remains notably underrepresented in mHealth interventions aimed at preventing childhood obesity.

To address these limitations, we developed the Good Start Matters mHealth Parenting Program, a theory-informed mHealth intervention designed to promote both individual parenting practices and collaborative coparenting strategies in the context of child eating, physical activity, and screen-time routines during early childhood. This protocol describes the randomized controlled trial (RCT) testing the efficacy of the Good Start Matters Parenting Program.

### Purpose and Hypothesis

The RCT described in this protocol evaluates the efficacy of an mHealth intervention aimed at improving parenting and coparenting practices (primary outcomes) and child health behaviors (secondary outcomes) in families with children aged 2.5-6 years. We hypothesize that families participating in the intervention will show significant improvements in parenting practices (increased structure and autonomy promotion, reduced control), coparenting agreement/consistency, and children’s health behaviors (eating, active and outdoor play, and screen-time) compared with control group families.

## Methods

### Study Design

**Figure 1:**
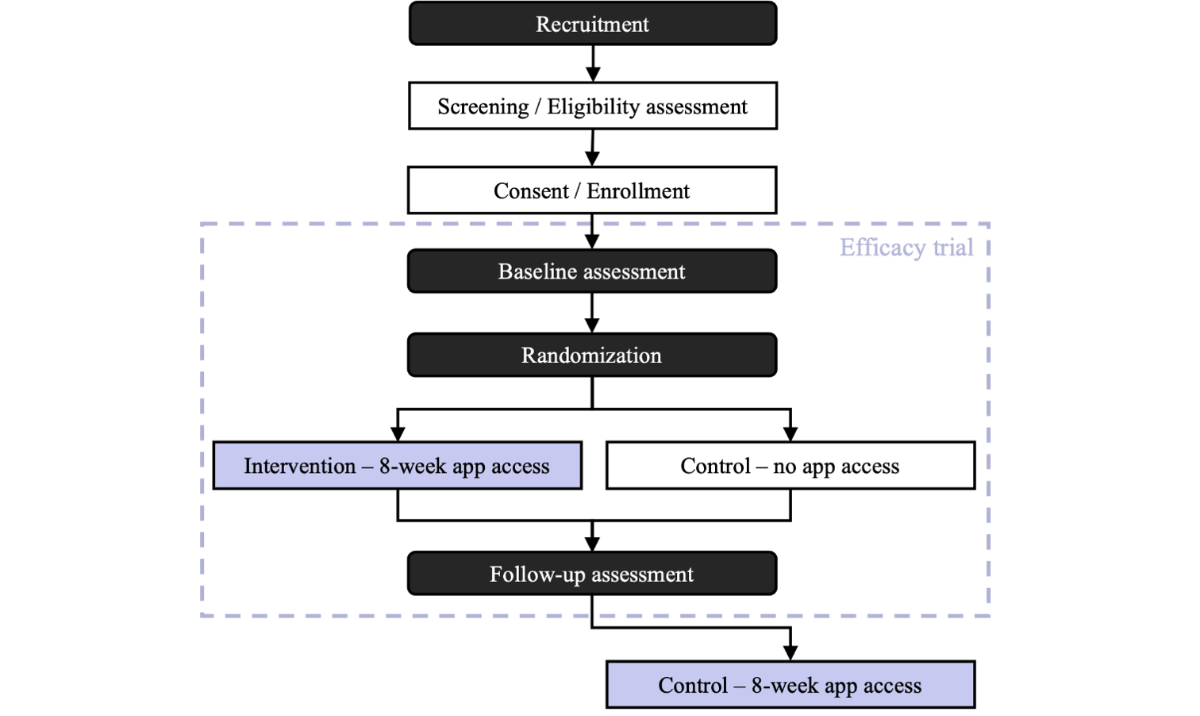
Flow diagram depicting the study’s methodology.

With an experimental design, this article presents the protocol of a 2-arm RCT evaluating the efficacy of the Good Start Matters mHealth Parenting Program; an 8-week intervention targeting positive parenting and coparenting practices and healthy child behaviors among British Columbian families with young children. Participating families will complete baseline assessments and then be randomized into an intervention or a waitlist control condition, with the former giving families access to the mHealth app immediately after baseline, and the latter providing families with app access after the follow-up assessment, approximately 2 months later (Figure 1).

### Ethical Considerations

This RCT was approved by the Research Ethics Board of the University of British Columbia (UBC, H18-01434), and all procedures were conducted in accordance with UBC’s ethical standards, as well as the 1964 WMA Declaration of Helsinki and its later amendments. The trial was prospectively registered in ClinicalTrials.gov in March 2023 (NCT05802160). This protocol has been described in compliance with SPIRIT (Standard Protocol Items: Recommendations for Interventional Trials [37]), and the trial will be conducted and reported in accordance with the CONSORT (Consolidated Standards of Reporting Trials [38]) and the CONSORT-EHEALTH (Consolidated Standards of Reporting Trials of Electronic and Mobile Health Applications and Online Telehealth) checklist for reporting electronic and mHealth applications [39]. All participants will be requested to read and sign an informed consent, which will explain the nature and consequences of participating in this study, prior to enrolling in the study. Participants will be able to either sign a web-based consent in REDCap (Research Electronic Data Capture [40]) using the E-Consent framework or on paper. Participation in this trial will be entirely voluntary, and withdrawal will be permitted at any time without consequence. In such cases, participants will decide whether data collected up to that point will be retained or destroyed. While no known risks to families are anticipated, the use of the app may prompt parents to reflect on or discuss their parenting and coparenting practices, which could cause some discomfort for some individuals. Therefore, a list of support resources will be provided to all participants. All data will be kept strictly confidential and anonymous, deidentified using unique participant codes, stored securely on encrypted servers at the University of British Columbia/BC Children’s Hospital Research Institute, and accessible only to the research team. Only deidentified findings will be disseminated through academic publications, conferences, and other media. Participants will receive a CAD $20 (US $15) gift card for each completed survey (baseline and follow-up). Those who complete both surveys will also be entered into a draw to win a CAD $100 (US $73) gift card, with 1 in 30 odds of winning.

### Recruitment

This RCT will recruit families through their childcare centers, leveraging the recruitment infrastructure of a parallel RCT, the Good Start Matters Appetite to Play Plus (ATP+) study [41], which targets group-licensed childcare centers across the Greater Vancouver and Victoria areas in British Columbia. The ATP+ study evaluates an implementation support intervention in childcare settings and does not overlap or interfere with this parenting program. Managers of childcare centers invited to ATP+ will be asked to assist with recruitment by displaying advertisement posters for the parenting RCT at their facilities and distributing information packages to parents (printed and over email). Parents can enroll in the parenting RCT through various methods; scanning a QR code provided on the poster or in the printed invitation letters, accessing a URL link sent via email, or completing and signing a paper consent form which will be returned to the childcare facility, where the research team will collect and enter the information into REDCap, hosted at the BC Children’s Hospital Research Institute.

### Participant Eligibility

Eligibility will be assessed via screening questions included at the beginning of the consent process. Eligible families will then finalize the consent process and receive an automated email with a link to complete the baseline assessment. Participating caregivers must include at least one parent with full or partial custody of their child (families with more than one child can enroll, but data on parent-child interactions and child health behaviors will only be collected for one of their children aged 2.5-6 years). A second caregiver (eg, biological parent, adoptive parent, step-parent, grand-parent) residing with the child most of the time and engaged in child-rearing responsibilities during the study period will also be eligible to enroll. To participate in the program, families will have to meet the following criteria:

- At least one of the caregivers must have primary or shared custody of the child.
- Caregivers must be able to read and understand English.
- Caregivers must each own a smartphone device supporting the app.
- Children should not currently be participating in a pediatric weight management or nutritional program.
- Children should not have severe dietary or physical restrictions that would prevent them from adhering to Canadian dietary and 24-hour Movement Guidelines.
- Children must be aged 2.5-6 years. Note that this range was selected to target a critical window in early childhood, as the years from age 2 to 5 are pivotal for consolidating healthy behaviors and sustaining developmental gains [2]. Evidence indicates that multicomponent interventions combining diet and physical activity are effective for preventing and treating overweight and obesity in children up to the age of 6 years [42,43]. The upper limit also reflects the recruitment context in childcare settings, where some children are slightly older than 5 years but share similar routines, developmental characteristics, and caregiving environments with younger peers, thereby maintaining the intervention’s ecological validity in this context.

### Sample Size

The sample size calculation for this RCT is based on the primary outcome: parenting practices. The study is designed to achieve 80% power at an α level of 0.05 to detect a group difference of 0.5 points on a 5-point Likert scale (10% change), assuming a SD of 0.8. This 0.5-point difference corresponds to an effect size of Cohen *d* of 0.63, calculated as the mean difference divided by the anticipated SD (ie, 0.5÷0.8=0.63). According to Cohen conventional thresholds, where small effect >0.2, moderate effect >0.5, and large effect >0.8 [44], a *d* of 0.63 represents a moderate effect size. The assumed SD of 0.8 is drawn from secondary data analyses [4,45] of the measurement study that developed the PAPP and FPP item banks, which are the instruments used in this study. Based on this calculation, a minimum of 41 families per group (82 families in total) is required [46]. To account for an anticipated 30% attrition rate and missing data, informed by a previous Canadian study engaging one caregiver per family in an mHealth lifestyle intervention for pediatric obesity [47], we estimate that at least 118 families will be needed for this RCT. Given the additional challenges associated with recruiting a second caregiver and, in particular, fathers [13], the target sample size specifically refers to 2-parent families, defined, for the purpose of this study, as a family where there are 2 caregivers participating in the trial. However, to promote inclusivity and support diverse family structures, one-parent families (ie, families with only one caregiver participating in the trial) will also be eligible to participate and be included in the overall sample (provided that they meet the eligibility criteria) without counting them toward the target sample of 118 two-parent families. Therefore, recruitment will stop once 118 two-parent families have been randomized, which may result in a larger overall sample size.

### Randomization

Families will be randomly assigned to intervention or waitlist control groups using a computer-generated randomization schedule created via Sealed Envelope [48]. Block randomization will be used with block sizes of 2, 4, and 6, and a 1:1 randomization ratio. The allocation schedule will be securely concealed within the randomization module of REDCap and will only be revealed after all participating caregivers in a family have completed the baseline assessments. To ensure integrity, research team members will not create, access, or modify the allocation schedule, as it will be fully computer-generated. Families will not be blinded to their group assignment because intervention group families will require immediate app access. Similarly, research team members will need to know the families’ group assignments to register them on the mHealth platform and follow up with them if technical issues arise. However, the randomization group will remain concealed from researchers during the data cleaning and analysis stage to minimize bias.

### The Good Start Matters mHealth Parenting Program

#### Theoretical Underpinning

The Good Start Matters mHealth Parenting Program will be heavily grounded in SCT [49,50], while also integrating critical constructs from Systemic and Structural Family Theories (SSFTs) [16,51] to enhance parenting and coparenting practices in support of children’s healthy behaviors. SCT highlights the interplay between personal, behavioral, and environmental influences that shape human behavior, recognizing the individual’s abilities to alter environments to suit their purposes (a concept known as reciprocal determinism [49,50]). Similarly, SSFT views the family as a complex system; a deeply connected, changing collection of parts, subsystems, and family members, where each member has a known purpose or function [52]. SSFT recognizes that the behaviors of one member impact the entire system, and change requires addressing the behaviors of all individuals that are part of the system [52]. Therefore, this mHealth intervention will focus on supporting multiple caregivers within the family with strategies to create health-promoting home environments that enable parental use of best practices to promote children’s health and well-being.

This intervention will leverage SCT’s focus on outcome expectation [49,50] by linking specific parenting behaviors to measurable child health outcomes, helping parents visualize the long-term benefits of their efforts over short-term challenges. In addition, it will encourage parents to communicate with their child about the importance of adopting healthy lifestyle behaviors. To help parents navigate barriers, the program will incorporate strategies such as self-monitoring, goal-setting, feedback, and social support to build the practical self-regulatory skills necessary for changing and sustaining behavior change as described in the next section. Through this program, parents will also be encouraged to use autonomy-promoting parenting practices thought to facilitate self-regulatory skills in children [7] and support their development toward becoming adults capable of making healthy decisions.

Self-efficacy, another cornerstone of SCT [49,50], is thought to be essential for driving behavior change. This mHealth intervention will use diverse approaches (eg, verbal encouragement, peer-modeling) to enhance both parents’ and children’s confidence in their ability to adopt and sustain positive practices and healthy behaviors. Moreover, SCT also emphasizes collective efficacy, the shared belief in a group’s ability to achieve desired outcomes [49,50]. Similarly, SSFT highlights the importance of parents’ confidence in their leadership roles within the family hierarchy, while also promoting an appropriate family structure [16] (defined as an invisible set of functional demands that organize the ways in which family members interact, including the subsystems, boundaries, hierarchies, and coalitions [16,52,53]). A critical subsystem within the family is the coparenting subsystem. As such, this mHealth intervention will engage both parents in activities that promote collaborative teamwork, shared goal-setting, collective responsibility, and reinforcement of each other’s efforts to strengthen the coparenting subsystem’s capacity to create a cohesive home environment. Through topics such as environmental control, consistent parenting practices, rule-setting, and operating conditioning, this intervention aims to empower coparents to establish stable family hierarchies [16,53] where they can confidently guide their children while continuing to promote their psychological autonomy.

Observational learning is another SCT mechanism integrated into this intervention [49,50]. Coparents will model and learn from each other’s parenting practices, observing the outcomes in their child and adapting their practices accordingly. This approach is expected to be effective because parents within the family likely face similar challenges, making peer-modeling relatable and actionable. Parental healthy lifestyle modeling will also be encouraged to help children engage in healthy behaviors [7].

Finally, recognizing that behavior change requires supportive environments [49,50], the program will provide facilitation strategies to remove potential barriers, as suggested in SCT. Program resources such as educational materials, step-by-step guides to implement positive practices, case scenarios, family-friendly recipes, suggestions for active play activities, and effective communication tips will help families address potential structural challenges (both physical and relational), make changes more accessible and sustainable, and facilitate children’s healthy lifestyles as they grow by modifying their home environment.

#### Program Design and Behavior Change Techniques Included

##### Overview

The program was initially pilot-tested with 2 families with toddlers and preschoolers, providing early insights into its feasibility and acceptability. A follow-up qualitative study, approved by the Research Ethics Board at UBC, will further explore participants’ perspectives on the intervention, including its perceived efficacy and feasibility.

**Figure 2:**
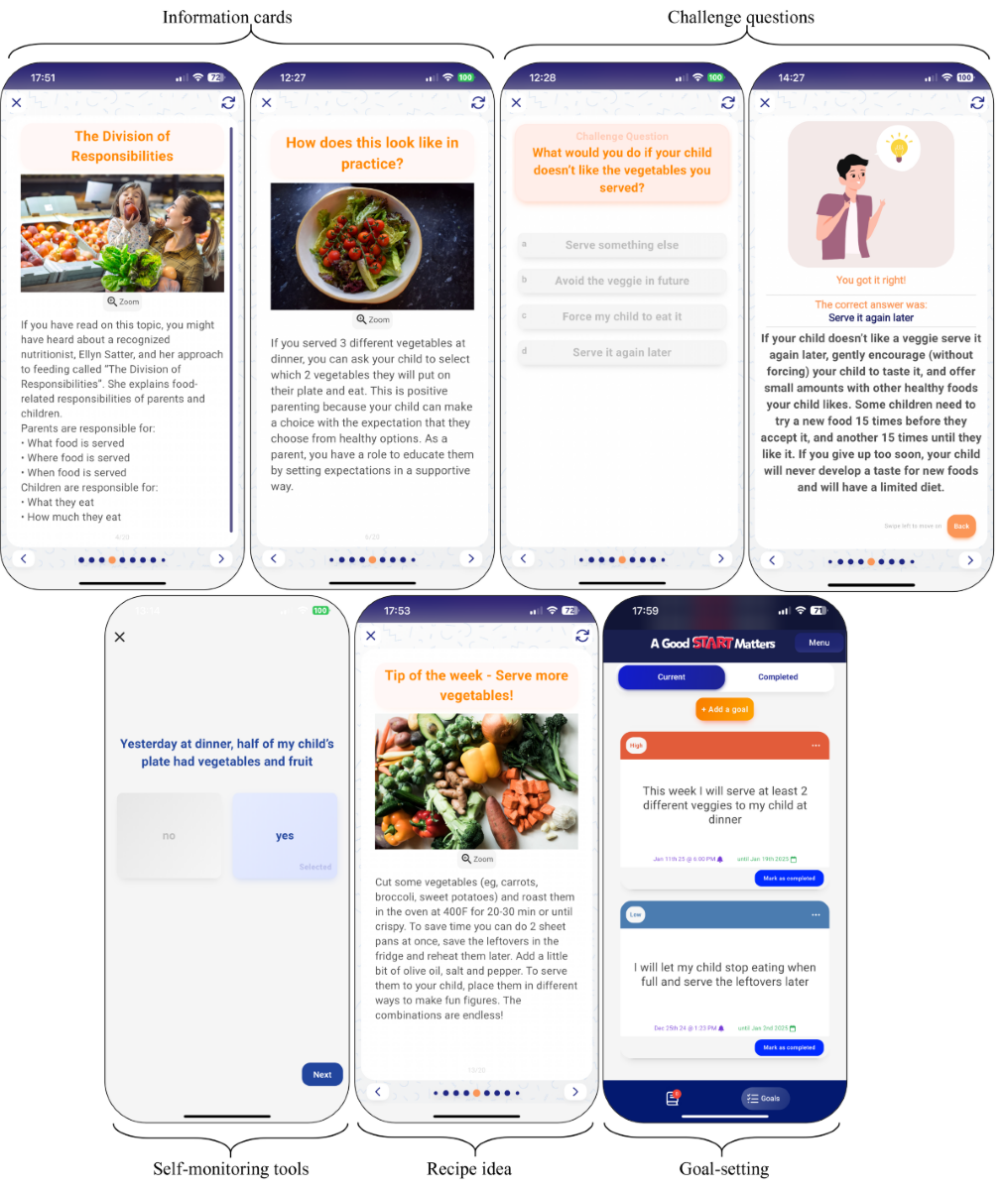
Screenshots of the Good Start Matters mHealth (mobile health) Parenting Program focused on feeding practices.

**Figure 3:**
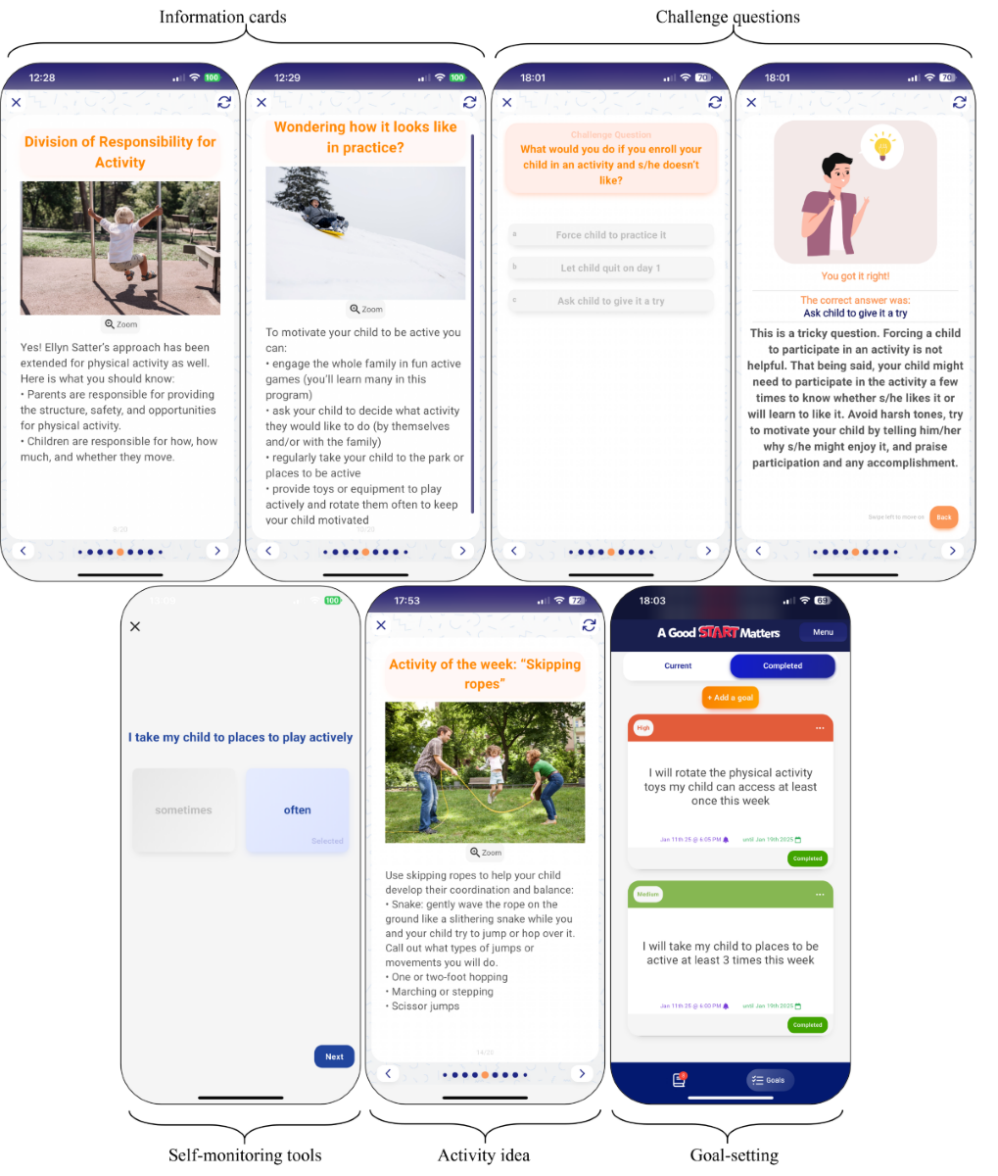
Screenshots of the Good Start Matters mHealth (mobile health) Parenting Program focused on physical activity.

To facilitate changes at both individual and family levels, this intervention will integrate a variety of evidence-based BCTs [54] across 9 modules delivered weekly over 8 weeks (see screenshots of the intervention in Figures 2 and 3). Each weekly module will be designed to take approximately 20 minutes to complete, including reviewing the core educational content and interactive elements. Additionally, caregivers will be encouraged to engage in independent activities such as goal setting, reflection exercises, and communication with their coparent, which are not counted toward the estimated 20-minute time commitment.

Reminders and notifications will be sent to participants every time a new module is released. Specifically, each week, participants will receive an automated email reminder informing them that a new module has been released. In addition, when participants set a goal within the app, they will be prompted to configure in-app notifications, which they may choose to enable to receive reminders related to their goal completion or review.

The text below provides a general description of each module, and illustrates examples of alignment between the program’s goals and evidence-based BCTs based on the taxonomy of Michie et al [54].

##### Module 0: Welcome

This introductory module will offer a general overview of the app’s functionality and features, outline the content to be covered, and provide access to reliable information sources and external services for more specialized needs.

##### Module 1: Get Ready to Make Changes

This module will introduce parents to foundational concepts, goals, and expectations for young children. Recommendations will be related to healthy eating, active play, and screen time based on the Canadian Food Guide [55] and the Canadian 24-hour Movement Guidelines [56]. This module will also highlight the importance of developing fundamental movement skills such as balancing, locomotion, and coordination to support engagement in more complex physical activities later in life [57,58]. Using self-assessment tools integrated into the app, parents will evaluate their child’s current health habits, identify areas for improvement, and set goals.

Examples of BCTs [54] included in this module are as follows:

- Shaping knowledge: Structure of a healthy plate; screen time, and active play guidelines; what are fundamental movement skills (balancing, locomotion, and coordination).
- Natural consequences: Benefits of promoting healthy eating, active habits, and fundamental movement skills.
- Comparison of outcomes: Citing Canada’s Food Guide [55], and 24-hour Movement Guidelines [56] as credible sources.
- Monitoring and feedback: Evaluation of the extent to which the child meets health behavior guidelines.
- Goals and planning: To serve a healthy plate and facilitate active play.
- Rewards and threats: Encouragement to use reward charts with the child.

##### Module 2: Divide Responsibilities

This module will focus on the division of responsibilities in dietary and activity contexts, drawing from Ellyn’s approach [59,60]. Parents will learn to differentiate between their role in providing healthy options and their child’s role in making decisions from the healthy options offered (“the parent provides, the child decides”). Practical examples will demonstrate how to implement this approach in real-life situations, while challenge questions will help parents anticipate and apply strategies in daily life. Self-assessment tools will encourage reflection on how well parents currently provide healthy opportunities for their child to make age-appropriate decisions, and recommended goals will emphasize both exposure to healthy options and child decision-making.

Examples of BCTs [54] included in this module are as follows:

- Shaping knowledge: Division of Responsibilities in eating and activity; exposure to healthy foods; facilitation and support for active play; reducing pressure and forceful practices.
- Natural consequences: Benefits of providing options within boundaries and encouraging age-appropriate decisions.
- Generalization of target behavior: Division of responsibility concept initially taught in the nutrition context and then extended to active play situations.
- Comparison of outcomes: Citing Ellyn’s Division of Responsibility approach as a credible source [59,60].
- Monitoring and feedback: Evaluation of the extent to which the parent provides healthy opportunities and challenge scenarios on how to deal with a picky eater.
- Goals and planning: To serve 2+ veggies at dinner; engage in suggested fundamental movement skills activities; or take children to places to be active.
- Rewards and threats: Evaluative feedback to parents after completing self-monitoring tools and challenge questions.
- Self-belief: Encouragement of gentle parental persuasion to promote child engagement in healthy behaviors.

##### Module 3: Provide Opportunities

Centered on stimulus control [61,62], this module will emphasize the role of parents in modifying the home environment to promote healthy habits, such as managing food accessibility, removing screens from bedrooms, rotating active play equipment, and offering opportunities for both organized and unstructured physical activities. Parents will critically examine their home environment to identify and modify unhealthy stimuli, aiming to “make the healthy option the easy choice.” Self-assessment tools will evaluate how well the home environment supports healthy behaviors, challenge questions will address common barriers, and recommended goals will focus on increasing the availability of healthy options at home.

Examples of BCTs [54] included in this module are as follows:

- Shaping knowledge: Controlling stimulus and environmental antecedents of unhealthy habits at home, exposure/availability of healthy foods, and facilitation of active play.
- Natural consequences: Benefits of exposure to healthy environments.
- Antecedents: Adding objects and modifying the home environment to facilitate adoption of healthier behaviors.
- Generalization of target behavior: Stimulus control and repeated exposure concepts initially taught in the nutrition context and then extended to active play.
- Monitoring and feedback: reflection on what the child’s home environment stimulates and a scenario on how to deal with a child rejecting a specific sport class.
- Goals and planning: To cook healthy plate meals; plan healthy snacks ahead of time; rotate active play toys; or look for sport/dance classes.
- Rewards and threats: Evaluative feedback to parents after completing self-monitoring tools and challenge questions.
- Social support: Encouragement of parental practical, emotional, and unspecified support for children to engage in healthy behaviors.
- Associations: Prompts/cues in the environment to influence parent and child behaviors; remove rewarding stimulus at home.

##### Module 4: Engage Your Family

This module will highlight the role of family interactions in fostering healthy lifestyle behaviors. Parents will learn to model healthy habits, such as eating nutritious meals and staying active, and to establish family routines that support positive modeling. It will emphasize the importance of family meals and shared activities, providing practical tips for making these experiences enjoyable. Self-assessments will evaluate current family routines and modeling behaviors, while recommended goals will focus on family-centered activities like regular family meals and fun active games together.

Examples of BCTs [54] included in this module are as follows:

- Shaping knowledge: How to establish family-centered routines and create opportunities for modeling family-based interactions.
- Natural consequences: Benefits of family-based activities and positive/healthy role modeling.
- Antecedents: Advice to maximize quality family time, modeling, and positive experiences.
- Generalization of target behavior: Healthy plate concept initially focused on child meals, further extended to the whole family; focus on creating regular family meals and active time.
- Monitoring and feedback: Evaluation of current family activities and the extent to which parents model healthy habits.
- Goals and planning: To coparticipate in active play; eat a healthy plate in front of the child; or have family meals.
- Rewards and threats: Evaluative feedback to parents after completing self-monitoring tools and challenge questions.
- Identity: Framing parents as key role models.
- Social support: Promotion of parental support (practical, emotional, and unspecified) to engage children in healthy behaviors.
- Self-belief: Encouragement of gentle parental persuasion to promote child engagement in healthy behaviors.
- Associations: Ensuring fun/enjoyable games are part of family physical activities and mealtimes.

##### Module 5: Create Routines

This module will emphasize the importance of structure and boundaries in fostering healthy routines. Parents will learn to establish consistent mealtimes, manage screen time, and create predictable schedules that support healthy habits. The module will highlight the need to place limits on less healthy behaviors, such as unhealthy food intake and excessive screen time, while encouraging active play without unnecessary restrictions to support the development of fundamental movement skills. Practical tips will include creating screen-free zones, setting rules around sugary treats, promoting outdoor exploration, and prioritizing family-centered activities. Self-assessments will help parents evaluate their current boundaries, and recommended goals will include pairing limits with education to encourage outdoor play and consistent meal routines.

Examples of BCTs [54] included in this module are as follows:

- Shaping knowledge: Creating routines, setting boundaries and limits in foods and screen time, and rethinking risky play boundaries.
- Natural consequences: Benefits of predictable environments; eating without distractions; consequences of highly processed foods intake, and risky play restrictions.
- Repetition and substitution: Focus on creating regular family meals and active time; replacing screen time during meals with fun-fact activities.
- Monitoring and feedback: Evaluation of the extent to which parents have boundaries and regular routines at home.
- Goals and planning: To eat meals and snacks at regular times; limit treats and sugary drinks or screen time; or let the child explore their limits at the playground.
- Rewards and threats: Evaluative feedback to parents after completing self-monitoring tools and challenge questions.

##### Module 6: Give Positive Feedback

Based on operant conditioning principles [63] (“behaviors depend on their consequences”), this module will teach parents how to respond to their child’s actions. Practical tips and examples will demonstrate how to use differential attention to reinforce only healthy behaviors with effective feedback methods [61,62], including verbal praise, nonfood/nonscreen rewards, physical gestures, and second- or third-hand compliments engaging multiple caregivers. Self-assessments will help parents reflect on whether they are reinforcing healthy behaviors or unintentionally encouraging negative ones, challenge questions will provide practice scenarios (eg, managing food-related tantrums), and recommended goals will emphasize using some of the feedback techniques provided.

Examples of BCTs [54] included in this module are as follows:

- Shaping knowledge: How to respond to child health behaviors to increase/decrease the likelihood of occurrence; how to deal with tantrums.
- Natural consequences: Consequences of different types of parental immediate responses to child behaviors (ie, reinforcing, punishing, or ignoring).
- Monitoring and feedback: Evaluation of the extent to which parents reinforce healthy or unhealthy child behaviors.
- Goals and planning: To provide positive feedback after healthy eating or small rewards for active behaviors, or use second-hand praising techniques.
- Rewards and threats: Evaluative feedback to parents via self-monitoring tools and challenge questions; encouragement of incentives and reward systems for children based both on effort and outcomes.
- Social support: Encouragement of parental practical, emotional, and unspecified support for children to engage in healthy behaviors.
- Self-belief: Encouragement of gentle parental persuasion to promote the child’s active role and engagement in healthy behaviors.
- Associations: Removing rewards for unhealthy behaviors.

##### Module 7: Involve Your Child

This module will focus on providing guidance and education on how to involve children in age-appropriate decision-making to promote psychological autonomy. Parents will learn strategies such as involving their child in simple meal preparation tasks (eg, washing vegetables, arranging ingredients) and teaching physical skills (eg, biking, kicking a ball) paired with gentle encouragement to help children understand the benefits of healthy choices, read their body signals, and feel motivated to participate in physical activities without pressure. Self-assessment tools will help parents reflect on how much autonomy their child currently has, and recommended goals will focus on empowering children to make simple decisions within a range of only healthy options.

Examples of BCTs [54] included in this module are as follows:

- Shaping knowledge: How to promote children’s autonomy and skills to make healthy choices as they grow through age-appropriate decisions, education, and guidance.
- Natural consequences: Benefits of supporting the child’s decision-making process within boundaries.
- Generalization of target behavior: Child involvement in simple tasks and decision-making was first presented in the nutrition context and then extended to the activity context.
- Monitoring and feedback: Evaluation of the extent to which the child can decide on their own activity and eating habits within boundaries.
- Goals and planning: To respect children’s satiety cues; involve them in simple meal preparation; or ask about preferred physical activities to try.
- Rewards and threats: Evaluative feedback to parents via self-monitoring tools and challenge questions.
- Social support: Encouragement of parental support (practical, emotional, and unspecified) to engage children in healthy behaviors.
- Self-belief: Encouragement of parental persuasion to promote the child’s active role and engagement in healthy behaviors.

##### Module 8: Work as a Team

This module will address the importance of positive coparenting practices to strengthen the coparenting subsystem and create a consistent and supportive environment. Parents will be encouraged to collaborate with other key caregivers to agree on goals, expectations, and practices, facilitated by effective communication and cooperative decision-making tips. Self-assessment tools will evaluate alignment in coparenting practices, and recommended goals will emphasize partnership and collaboration in child-rearing tasks related to lifestyle habits.

Examples of BCTs [54] included in this module are as follows:

- Shaping knowledge: How to engage in positive coparenting practices; effective communication; cooperative and consistent practices around child lifestyle habits.
- Natural consequences: Consequences of disagreements and conflicts in front of the child; inconsistent approaches and lack of structure.
- Monitoring and feedback: Evaluation of the extent to which coparents’ expectations, goals, and practices about the child’s behaviors align.
- Goals and planning: To discuss goals and expectations for the child and approaches to meet these goals; praise the coparent for using practices they have agreed on; or provide structure at home.
- Rewards and threats: Evaluative feedback to parents via self-monitoring tools and challenge questions; promotion of social reward to coparent for engaging in an agreed-on parenting practice.
- Social support: Coparents are encouraged to seek help from and support each other to engage in positive parenting practices.

##### Module 9: Recap and Commit

The final module will summarize the program’s content and key takeaways. It will encourage parents to reflect on the things they have accomplished, the resources and skills they can use, and commit to continue using health-promoting parenting and coparenting practices.

Examples of BCTs [54] included in this module the following:

- Shaping knowledge: Recap on health behavior guidelines regarding nutrition, active play, and screen time; recommended autonomy-promoting and structured practices; division of responsibility in nutrition and activity; importance of family-centered activities, and joint family management.
- Monitoring and feedback: Review of progress made; existing strengths, resources, and skills to continue using positive parenting and coparenting practices.
- Goals and planning: Parents asked to make statements and commitments to maintain existing or use new positive parenting and coparenting practices.
- Identity: Framing parents and caregivers as key role models.
- Social support: Encouragement of parental social support for children to engage in healthy behaviors.
- Self-belief: Focus on previous successes in using positive parenting and coparenting practices.

### Measures

#### Overview

Data collection will occur at baseline and follow-up (approximately 2 months after randomization) for all variables listed below except for sociodemographic variables, which will be evaluated only at baseline. Participants will complete all surveys online using REDCap.

#### Primary Outcomes

Parenting practices related to nutrition and activity will be measured with items modeled after the validated food [64] and physical activity [65] parenting item banks designed for 5- to 12-year-olds, with some questions adapted for younger children (eg, the original item asks “how often do you have your child help prepare dinner meals”), while the adapted item will ask “how often do you involve your child in simple food tasks when preparing meals (eg, help wash vegetables, arrange the vegetables). Both item banks were informed by parenting and obesity experts and have been evaluated for construct validity (ie, designed to capture the 3 parenting domains of control, structure, and autonomy promotion) related to nutrition and active play. These item banks have shown strong psychometric properties, including measurement invariance by parent gender, making the comparisons valid between mothers and fathers, and Cronbach α ranging from 0.67 to 0.96 [64-67]. Additional items on food, physical activity, and media parenting practices were developed for this study to align with the intervention content. Accordingly, we will assess and report on the psychometric properties of the parenting measurement tools used in this RCT, focusing on construct validity (via factor analysis) and reliability (via Cronbach α). Reassessing these properties in the final sample will provide a more accurate evaluation of the validity and reliability of the measures applied in this trial.

Coparenting practices will be evaluated with items adapted from the Agreement subscale of the Coparenting Relationship Scale [17], designed for children aged 6 months to 3 years and further used with children up to 7 years old [18,68]. The scale shows adequate psychometric properties, including construct validity and reliability (Cronbach α ranging from 0.67 to 0.74). The questions will inquire about the level of agreement between key caregivers regarding their expectations, goals, approaches, and conceptions about their child’s lifestyle behaviors. Additionally, coparenting practices will be examined in 2-parent families by comparing the parenting practice scores of both parents/caregivers within the family.

#### Secondary Outcomes

Child eating behaviors will be evaluated with the Child Eating Behavior Questionnaire [69], examining child emotional overeating, food responsiveness, food fussiness, and satiety responsiveness. This instrument, designed for children aged 2-9 years, has been extensively used, showing adequate psychometric properties, including Cronbach α ranging from 0.72 to 0.91 [69]. In addition, parents will be asked to report on the number of days per week that their child consumes key food markers (ie, fruit and vegetables, fruit juice and sugary drinks, sweet and salty treats), with questions adapted from the Canadian Community Health Survey [70] and the Center for Diseases Control’s Behavioral Risk Factor Surveillance System Questionnaire [71] and National Youth Physical Activity and Nutrition Study [72].

Child active and outdoor play will be measured with questions from the instrument designed by Burdette et al [73] for young children. The questions inquire about the number of minutes per day and the number of days per week that the child engages in active and outdoor play on a regular weekday and weekend day.

Children’s screen time will be measured by inquiring about the number of days in the previous week that children spent more than 1 hour/day in front of screens, when counting all screen-media devices (eg, TV, computer, tablet, and smartphone).

#### Covariates

Covariates will include child-level, parent-level, and family-level variables that may have an influence on family dynamics, parenting practices, and child health behaviors. Sociodemographic questions (some created for this study and others adapted from the Canadian Community Health Survey [70] and General Social Survey [74]), will cover child and parent age and sex, parent gender, marital status, education, employment status, cultural/ethnic background, household income, number of households where the child lives in, number of children living at home and their age group, number and type of family members in the household, and familial relationship between participating coparents. In addition, we will evaluate the division of responsibilities between key caregivers regarding child-rearing tasks (eg, grocery shopping, cooking, cleaning, transport, and education) with questions adapted from Musick et al [75].

#### Intervention Group and Exposure/Adherence

The intervention group will be assessed based on randomization as a binary variable (intervention vs control). Exposure/adherence to the mHealth intervention will be measured with app-analytics data collected with the platform used to deliver the intervention (ie, Pathverse). Pathverse is a no-code app builder platform that supports mHealth research [76,77], making it easy for researchers to create engaging interventions with preprogrammed features (eg, goal setting, monitoring, feedback) that are known to support behavior change [54]. As Pathverse was developed for research, it collects extensive app analytics data, which can then be examined to assess how many and which components of the intervention the users engaged with. Using these data, adherence will be operationalized as the number of modules completed, self-monitoring tools completed, and goals set and completed.

### Analyses

Descriptive statistics will be used to summarize the distribution of the data. Baseline comparisons between groups will be conducted using the Student *t* tests or chi-square tests, as appropriate, for continuous and categorical demographics, primary, and secondary outcomes. These comparisons will assess the extent to which differences remain between groups at baseline despite randomization. To evaluate the effect of the intervention, intention-to-treat multilevel mixed-effects models will be used. These models will be linear, ordered, or logistic, depending on the characteristics of the data and whether transformations are required. The models will examine the effects of the intervention on each key primary and secondary outcome, incorporating an interaction term between time point (baseline or follow-up) and randomization group (intervention or control). These multilevel analyses will account for the nested structure of the sample and the longitudinal nature of the data, with parents nested within families and repeated measures over time. Individual- and family-level covariates will be included in the models as appropriate. Secondary analyses will assess whether changes in parenting and coparenting practices, and the interaction between these two, predict changes in child health behaviors using multilevel and structural equation modeling. Additionally, dose-response sensitivity analyses with mixed-effect models will evaluate the relationship between the level of exposure/adherence to the intervention (measured via app analytics) and the magnitude of changes in parenting and coparenting practices and child health behaviors. If deemed necessary, imputation techniques will be applied to address missing data. In such a case, the specific imputation method (eg, multivariate multiple imputation, chained equations) will be selected based on variable types and patterns of missingness detected in the data.

## Results

Recruitment was conducted between November 2023 and January 2025, and data collection occurred from November 2023 through April 2025. In total, 121 two-parent families and 62 one-parent families were enrolled. Since May 2025, and as of October 3, 2025, the study has been in the data cleaning and analysis phase, which is expected to be completed by December 2025. Study findings will be reported in 2026, with planned publication and updates to the ClinicalTrials.gov registry. Results will also be submitted to peer-reviewed journals and disseminated at academic conferences.

## Discussion

### Anticipated Principal Findings

This RCT is designed to assess the efficacy of the Good Start Matters mHealth Parenting Program in enhancing parenting and coparenting practices and promoting healthier child behaviors related to lifestyle habits. We anticipate that families receiving the intervention will demonstrate greater use of structured and autonomy-promoting parenting and lower use of controlling practices, stronger coparenting agreement/consistency, and healthier child behaviors, including eating habits, active and outdoor play, and screen time, compared with families in the control group.

### Comparison to Prior Work

Family-based behavioral interventions remain the gold standard for preventing and addressing childhood obesity [25,26]. However, many in-person programs predominantly recruit a single caregiver (usually mothers), resulting in limited engagement from fathers or other key caregivers within the family system [13]. These interventions also tend to focus primarily on child outcomes [78], often overlooking important familial mediators such as parenting and coparenting practices, which play a central role in shaping children’s health behaviors. In addition, in-person programs face well-documented barriers to adherence and attendance, including financial costs, scheduling conflicts, transportation issues, and other logistical and organizational challenges [78-81].

mHealth interventions offer a promising alternative to address some of these limitations [79], particularly by creating more opportunities for fathers to participate (a strategy recommended for family-based interventions in other child health domains [82]). Beyond their convenience, mHealth platforms can reduce structural barriers such as travel distance, scheduling conflicts, and limited childcare by enabling participation from home or other preferred locations; an aspect parents value for allowing them to integrate sessions into daily routines, choose times that work best for them, and avoid the stress associated with in-person attendance [27-29].

The Good Start Matters mHealth Parenting Program will build on these strengths, using a structured, theory-driven approach informed by SCT and SSFT, and incorporating evidence-based BCTs to equip parents with tools and skills to create a supportive environment for their children’s health and development. This approach is consistent with mHealth research showing that programs grounded in these frameworks tend to be more effective [32-34].

However, despite their promise, existing mHealth interventions face challenges similar to those observed with in-person programs [30-33]. Notably, there is a substantial lack of mHealth studies targeting and measuring parenting practices [27,30-32,34-36] and, to our knowledge, none has explicitly focused on coparenting dynamics (despite the well-established influence of the family system and WHO’s recommendations endorsing family-based approaches to childhood obesity prevention [25]). The Good Start Matters Parenting Program will address these gaps by targeting both individual parenting practices and coparenting dynamics, aiming to achieve sustainable improvements in both family- and child-level outcomes.

Another key distinction is the focus on early childhood. Most mHealth interventions in this field have focused on older children and adolescents [27,32,35,47], with limited attention to younger age groups. The decision to intervene early is supported by evidence showing that 34% of Canadian toddlers and preschoolers are at risk of overweight or obesity [83] and excess weight gain before puberty largely occurs by the age of 5 years [84], which makes the early years a critical intervention window. Taken together, this study will contribute to the growing mHealth literature by addressing critical gaps in caregiver engagement, coparenting, and early childhood prevention, offering a model that could inform the design of future family-based interventions.

### Strengths and Limitations

This study will have some limitations, including a short intervention period and the absence of long-term follow-up assessments, which may affect observations of sustained changes. Future research could incorporate longitudinal designs to better assess longer-term effects. Strengths of this study should also be noted, including the involvement of multiple caregivers and the evaluation of intervention effects across the family system. Moreover, the program’s concise, self-paced format, requiring less than 30 minutes per week, will provide flexibility for parents, potentially enhancing adherence.

### Conclusions

Overall, this study will provide critical insights into the potential of mHealth interventions to improve parenting and coparenting practices and promote healthier child behaviors during early childhood. By addressing gaps in father engagement and coparenting dynamics, the Good Start Matters mHealth Parenting Program has the potential to strengthen the foundation of family-centered interventions and set a new standard for systemic approaches in childhood obesity prevention. Findings will inform the development of scalable, inclusive mHealth strategies practical for real-world implementation and tailored to diverse family needs, ultimately advancing the field of child health promotion.

## Abbreviations

ATP+: Appetite to Play Plus
BCT: behavior change technique
CONSORT: Consolidated Standards of Reporting Trials
CONSORT-EHEALTH: Consolidated Standards of Reporting Trials of Electronic and Mobile Health Applications and Online Telehealth
mHealth: mobile health
RCT: randomized controlled trial
REDCap: Research Electronic Data Capture
SCT: social cognitive theory
SPIRIT: Standard Protocol Items: Recommendations for Interventional Trials
SSFT: Systemic and Structural Family Theory
UBC: University of British Columbia

## Data Availability

Data sharing is not applicable to this article as no datasets were generated or analyzed during this study.
